# The Beneficial Effects of Beta Blockers on the Long-Term Prognosis of Patients With Premature Atrial Complexes

**DOI:** 10.3389/fcvm.2022.806743

**Published:** 2022-02-16

**Authors:** Ting-Chun Huang, Po-Tseng Lee, Mu-Shiang Huang, Pin-Hsuan Chiu, Pei-Fang Su, Ping-Yen Liu

**Affiliations:** ^1^Institute of Clinical Medicine, College of Medicine, National Cheng Kung University, Tainan, Taiwan; ^2^Division of Cardiology, Department of Internal Medicine, National Cheng Kung University Hospital, College of Medicine, National Cheng Kung University, Tainan, Taiwan; ^3^Department of Statistics, College of Management, National Cheng Kung University, Tainan, Taiwan; ^4^The Center for Quantitative Sciences, Clinical Medicine Research Center, National Cheng Kung University Hospital, College of Medicine, National Cheng Kung University, Tainan, Taiwan

**Keywords:** premature atrial complex (PAC), arrhythmia, beta blocker, prognosis, atrial fibrillation, stroke

## Abstract

**Aims:**

Premature atrial complexes (PACs) have been reported to increase the risk of adverse cardiovascular outcomes. Beta blockers at low dosages may help to reduce PAC symptoms, but it is unclear whether they can improve long-term outcomes.

**Methods:**

Patients enrolled from a Holter cohort in a medical referral center were stratified into high-burden (≥100 beats/24 h) and low-burden (<100 beats/24 h) sub-cohorts, and propensity score matching between treatment groups and non-treatment groups was conducted for each sub-cohort.

**Results:**

In the high-burden sub-cohort, after propensity score matching, the treatment group and non-treatment group respectively had 208 and 832 patients. The treatment group had significantly lower mortality rates than the non-treatment group [hazard ratio (HR) = 0.521, 95% confidence interval (CI) = 0.294–0.923, *p* = 0.025], but there was no difference in new stroke (HR = 0.830, 95% CI = 0.341–2.020, *p* = 0.681), and new atrial fibrillation (HR = 1.410, 95% CI = 0.867–2.292, *p* = 0.167) events. In the low-burden sub-cohort, after propensity score matching, there were 614 patients in the treatment group and 1,228 patients in the non-treatment group. Compared to the non-treatment group, up to 40% risk reduction in mortality was found in the treatment group (HR = 0.601, 95% CI = 0.396–0.913, *p* = 0.017), but no differences in new stroke (HR =0.969, 95% CI = 0.562–1.670, *p* = 0.910) or atrial fibrillation (HR = 1.074, 95% CI = 0.619–1.863, *p* = 0.800) were found.

**Conclusions:**

Beta blockers consistently decreased long-term mortality in high-burden and low-burden patients. Interestingly, this effect was not achieved through reduction of new-onset stroke or AF, and further research is warranted.

## Introduction

Premature atrial complexes (PACs) are a common type of arrhythmic disturbance in the general population (1). PAC burden is known to be age-dependent, but is not associated with sex (2). Patients with PACs are at increased risk of stroke and mortality over the long term, either due to the burden of PAC itself, or to subsequent atrial fibrillation (AF) (3–6). We have previously revealed an association between higher PAC burden and higher risk of all-cause mortality and cardiovascular death (2), and PACs have been reported to be an important factor in the initiation and perpetuation of AF (7). The main focal mechanisms of PACs, including enhanced automaticity, early afterdepolarization, or delayed afterdepolarization, are highly related to adrenergic activation (7). As sympathetic antagonists, beta blockers are known to bring long-term benefits to a number of cardiovascular conditions, particularly heart failure, which involves a vicious cycle of sympathetic overactivation. Patients with high PAC burden are commonly prescribed with beta blockers, especially for symptomatic sufferers, but the long-term effects of beta blockers on cardiovascular outcomes remain unclear as yet. Recently, we found that a threshold of ≥100 beats/24 h could serve as a cutoff for the prediction of adverse cardiovascular outcomes in patients with PACs (2). Therefore, in this study, we aimed to investigate the effects of beta blockers on the long-term prognosis of patients with a high (≥100 beats/24 h) or low (<100 beats/24 h) burden of PACs by using propensity score matched treatment and non-treatment groups.

## Methods

### Databank

We conducted a retrospective cohort study, using the Cardiovascular Disease Databank from National Cheng-Kung University Hospital (NCKU) to enroll consecutive patients who had undergone 24-h Holter monitoring. The Databank has previously been validated (2), and contains the complete anonymized inpatient and outpatient electronic medical records of the NCKU cardiovascular department, including patients who had undergone invasive or non-invasive cardiovascular studies. Longitudinal data regarding patient demographics, symptoms, laboratory data, medications, and imaging studies from January 1, 2009 to July 1, 2020 were collected. The Databank was established upon data collected under the Artificial Intelligence with Deep Learning and Genes on Cardiovascular Disease study, which is registered in ClinicalTrials.gov (NCT03877614). This study was approved by an independent ethics committee (IEC) at NCKU (A-ER-107-149, A-ER-108-381), and was conducted in accordance with institutional and local regulations, Good Clinical Practice (GCP), and the Declaration of Helsinki. Patient informed consent was waived due to the retrospective nature of this study and the anonymization of patient data.

### Study Cohort

**Figure 1:**
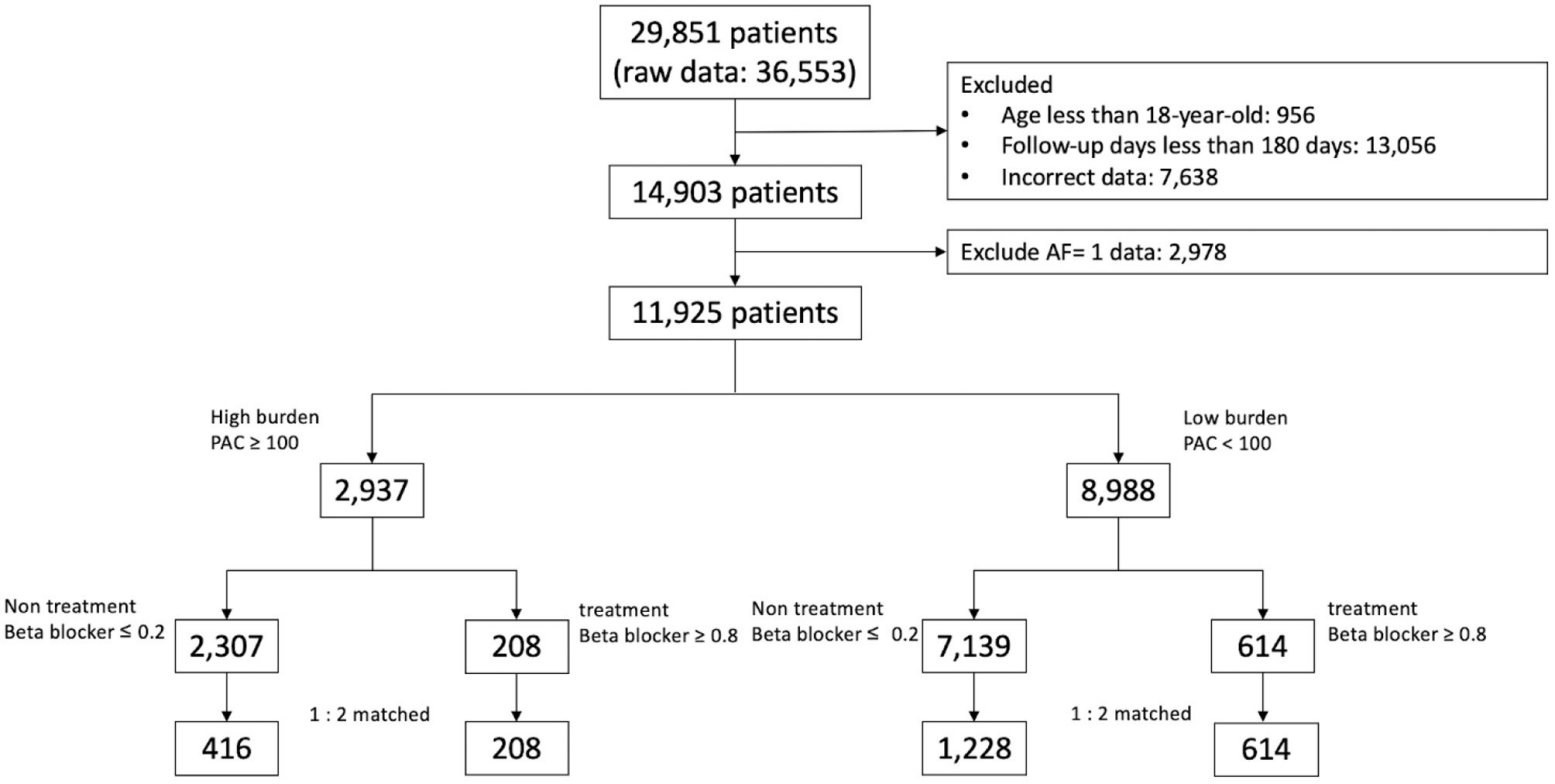
Study flow.

The Databank were consecutively analyzed from January 1, 2010 to August 31, 2019, and 29,851 Holter monitoring records from 36,553 patients were identified (Figure 1). Patients aged less than 18 years and who were followed up for less than 180 days were excluded. In total 2,978 patients who had a history of AF, as documented by electrocardiography or 24-h Holter recording before the indexed Holter examination were also excluded. For patients with repeated examinations, the earliest PAC burden and clinical information were used for analysis. The final study cohort included 11,925 patients, who were divided into high-burden (≥100 beats/24 h; *n* = 2,937) or low-burden (<100 beats/24 h; *n* = 8,988) sub-cohorts. In each sub-cohort, patients prescribed with regular beta blockers during ≥80% of the entire follow-up period were designated as the treatment group, while patients who never or seldomly (≤ 20% of the follow-up period) used beta blockers were designated as the non-treatment group, and were selected for analysis. The follow-up period was defined as 3 months before the index Holter examination until the last date on the hospital electronic medical record.

### Definition of Clinical Characteristics and Endpoints

The endpoints of this study respectively included all-cause mortality, new onset of stroke or transient ischemic accident (TIA), and new onset of AF. Baseline characteristics, comorbidities, and medications were all recorded on the date of enrollment. To ensure the accuracy of patient diagnoses, each variable was determined comprehensively based on the attending physician's manual input, laboratory results, corresponding treatment, and International Classification of Diseases (ICD) codes (2). All used medications were defined as regular prescriptions if they were given for more than 70% of the follow-up period. Mortality data was retrieved from the Collaboration Center of Health Information Application, Ministry of Health and Welfare in Taiwan, and further confirmed through linkage with the National Death Registry.

### 24-h Holter Monitoring

All patients were asked to follow their daily routines without any limitations during the recording period. A DR200/HE Holter (NorthEast Monitoring, Inc., Maynard MA, USA) with a frequency response of 0.05 to 70 hertz in 180 samples/second mode was used (2), with 7-lead placements to acquire triple-channel information: V5 (–, right manubrium; +, left anterior axillary line on the 5th rib), V1 (–, left of the manubrium; +, 2 cm right of the xiphoid process), and lead III (–, centered on the manubrium; +, left of the mid-clavicular line on the 5th rib). All recordings were analyzed using Holter LX Analysis (NorthEast Monitoring, Inc.), with the system programmed to automatically capture all ectopic beats or rhythmic disturbances. The recordings were reviewed by experienced technicians. PAC and PVC (premature ventricular complex) were defined as coupling interval <90 and <80% of the last coupling interval, respectively. Supraventricular and ventricular tachycardia episodes were defined as three or more consecutive supraventricular or ventricular beats, respectively, at a speed of more than 120 beats per minute. A PAC or a supraventricular event was considered when QRS duration was less than 120 milliseconds, unless aberrant morphology of QRS was detected, whereupon this would be considered as a PVC or ventricular tachycardia event. The cumulative number of PACs during the monitoring period was presented as beats/24 h. All arrhythmic episodes, unknown strips, and final formal 24-h Holter reports were reviewed and confirmed by qualified senior cardiologists.

### Statistical Analysis

Categorical variables were presented as frequencies and percentages, while continuous variables were reported as means with standard deviations (SD). Chi-squared test with two-tailed Fisher's exact test and Student's *t*-test were respectively used for intergroup comparison of categorical and continuous variables. In order to exclude all possible confounders in each group, propensity score matching between patients who regularly took beta blockers (treatment group) with patients who never or seldomly took beta blockers (non-treatment group) was conducted at a ratio of 1:2 for both high-burden and low-burden sub-cohorts, using a near-neighbor matching algorithm with caliper width of 0.2. The matched variables included age, sex, PAC burden, follow-up days, medical history (diabetes mellitus, dyslipidemia, hypertension, stroke, coronary artery disease, chronic kidney disease, heart failure, peripheral arterial disease, hypertrophic cardiomyopathy), and drug history (aspirin, angiotensin converting enzyme inhibitor/angiotensin receptor blocker, diuretics, P2Y12 inhibitor, warfarin, and non-vitamin K antagonist oral anticoagulants). Analysis of survival data was then conducted to evaluate the effect of beta blocker treatment. The primary and secondary endpoints were analyzed using the Cox-proportional hazard model. Univariate analysis of beta blocker use against endpoints was performed, and hazard ratios (HR) with 95% confidence intervals (CI) were calculated. In addition, cumulative event-rate curves were plotted, and the log-rank test was used to compare the survival distributions of treatment and non-treatment groups in each sub-cohort. All statistical tests were 2-sided, and *p* value <0.05 was considered to be statistically significant. All analyses were performed with R statistical software, version 3.6.3 for Windows.

## Results

### Baseline Characteristics of the Study Cohort

Baseline characteristics of patients in the high-burden and low-burden sub-cohorts are presented by beta blocker treatment in Tables 1, 2. In the high-burden sub-cohort, 2,307 and 208 patients were respectively included in the non-treatment and treatment groups. Mean follow-up was respectively 1,270.56 ± 923.82 days and 1,087.02 ± 935.48 days. No differences were noted in age, sex, and medical history, including stroke, coronary artery disease, heart failure, peripheral arterial disease, hypertrophy cardiomyopathy, use of non-vitamin K antagonist oral-anticoagulants or warfarin, PAC burden, and PVC (premature ventricular complex) burden (980.9 ± 3,313.5 in non-treatment group and 1,052.9 ± 3,609.8 in treatment group, *p* = 0.650). Patients in the treatment group had shorter follow-up duration, more comorbidities, including diabetes mellitus, dyslipidemia, hypertension, and chronic kidney disease, and took more medications (e.g. aspirin, P2Y12 inhibitors, and angiotensin converting enzyme inhibitors/angiotensin receptor blockers) than patients in the non-treatment group before propensity score matching. For patients in the low-burden sub-cohort, the treatment group (*n* = 614) was much older, underwent shorter follow-up (1,095.01 ± 1,033.18 days vs. 1,344.04 ± 995.22 days), had more comorbidities, including diabetes mellitus, hyperlipidemia, hypertension, coronary artery disease, heart failure, higher PAC burden, and used more medications (similar to those in the high-burden sub-cohort), than the non-treatment group (*n* = 7,139). However, treatment group had less PVC burdens (1,017.6 ± 4,705.9 vs. 903.0 ± 3,630.6, *p* < 0.001). After propensity score matching, no differences in any variables between the treatment and non-treatment groups of each sub-cohort were noted. Although we did not further match PVC burdens, no difference in PVC burden was found among high-burden sub-cohort (non-treatment group vs. treatment group = 980.9 ± 3,313.5 vs. 1,052.9 ± 3,609.8, *p* = 0.059). Small difference was noted among low-burden sub-cohort (969.9 ± 3,666.9 vs. 903.0 ± 3,630.6, *p* = 0.049). The study flow is presented in Figure 1.

**Table 1:** Demographic and clinical characteristics of patients with a high burden of PACs in the treatment and non-treatment groups before and after propensity score matching.

**Variables**	**Before matching**				**After matching**			
	**Non-Tx** **(*N* = 2,307)**	**Tx** **(*N* = 208)**	***p***	**SMD**	**Non-Tx** **(*N* = 416)**	**Tx** **(*N* = 208)**	***p***	**SMD**
Age, y, mean (SD)	70.65 (13.90)	69.18 (12.37)	0.143	0.111	68.71 (14.55)	69.18 (12.37)	0.689	0.035
Male, *N* (%)	1,147 (49.7)	98 (47.1)	0.518	0.052	181 (43.5)	98 (47.1)	0.442	0.072
Follow-up days, mean (SD)	1,270.56 (923.82)	1,087.02 (935.48)	0.006	0.197	1,057.33 (849.33)	1,087.02 (935.48)	0.691	0.033
PACs, mean (SD)	2,540.70 (5,961.64)	3,202.32 (6,886.76)	0.131	0.103	3,293.86 (8,308.50)	3,202.32 (6,886.76)	0.891	0.012
HTN, *N* (%)	1,402 (60.8)	164 (78.8)	<0.001	0.402	323 (77.6)	164 (78.8)	0.811	0.029
DM, *N* (%)	608 (26.4)	69 (33.2)	0.041	0.150	119 (28.6)	69 (33.2)	0.280	0.099
Dyslipidemia, *N* (%)	1,078 (46.7)	131 (63.0)	<0.001	0.331	267 (64.2)	131 (63.0)	0.837	0.025
HF, *N* (%)	340 (14.7)	29 (13.9)	0.835	0.023	41 (9.9)	29 (13.9)	0.164	0.126
CAD, *N* (%)	341 (14.8)	41 (19.7)	0.072	0.131	73 (17.5)	41 (19.7)	0.583	0.056
PAOD, *N* (%)	73 (3.2)	2 (1.0)	0.115	0.155	3 (0.7)	2 (1.0)	>0.999	0.026
Stroke, *N* (%)	237 (10.3)	20 (9.6)	0.857	0.022	34 (8.2)	20 (9.6)	0.651	0.051
CKD, *N* (%)	729 (31.6)	45 (21.6)	0.004	0.227	73 (17.5)	45 (21.6)	0.263	0.103
HCM, *N* (%)	48 (2.1)	9 (4.3)	0.066	0.128	13 (3.1)	9 (4.3)	0.591	0.063
Aspirin, *N* (%)	259 (11.2)	50 (24.0)	<0.001	0.341	93 (22.4)	50 (24.0)	0.711	0.040
P_2_Y_12_ inhibitor, *N* (%)	95 (4.1)	27 (13.0)	<0.001	0.321	44 (10.6)	27 (13.0)	0.449	0.075
Warfarin, *N* (%)	18 (0.8)	2 (1.0)	1.000	0.020	5 (1.2)	2 (1.0)	>0.999	0.023
NOAC, *N* (%)	21 (0.9)	4 (1.9)	0.296	0.086	7 (1.7)	4 (1.9)	>0.999	0.018
ACEi/ARB, *N* (%)	207 (9.0)	39 (18.8)	<0.001	0.286	69 (16.6)	39 (18.8)	0.575	0.057

*AAD, antiarrhythmic drug; ACEi/ARB, angiotensin-converting enzyme inhibitor/angiotensin receptor blocker; AF, atrial fibrillation; CAD, coronary artery disease; CKD, chronic kidney disease; DM, diabetes mellitus; HCM, hypertrophic cardiomyopathy; HF, heart failure; HTN, hypertension; NOAC, novel oral anticoagulant; PAOD, peripheral arterial occlusive disease; PAC, premature atrial complex; SMD, standardized mean difference; Tx, treatment*.

**Table 2:** Demographic and clinical characteristics of patients with a low burden of PACs in the treatment and non-treatment groups before and after matching.

**Variables**	**Before matching**				**After matching**			
	**Non-Tx** **(*N =* 7,139)**	**Tx** **(*N =* 614)**	***p***	**SMD**	**Non-Tx** **(*N =* 1,228)**	**Tx** **(*N =* 614)**	***p***	**SMD**
Age, y, mean (SD)	56.55 (15.99)	61.01 (12.72)	<0.001	0.309	61.70 (14.49)	61.01 (12.72)	0.317	0.051
Male, *N* (%)	3,218 (45.1)	296 (48.2)	0.146	0.063	608 (49.5)	296 (48.2)	0.633	0.026
Follow-up days, mean (SD)	1,344.04 (995.22)	1,095.01 (1,033.18)	<0.001	0.246	1,045.11 (900.22)	1,095.01 (1,033.18)	0.286	0.051
PACs, mean (SD)	19.45 (22.67)	21.36 (23.18)	0.046	0.083	22.14 (23.49)	21.36 (23.18)	0.499	0.034
HTN, *N* (%)	3,042 (42.6)	441 (71.8)	<0.001	0.618	942 (76.7)	441(71.8)	0.026	0.112
DM, *N* (%)	1,446 (20.3)	173 (28.2)	<0.001	0.186	359 (29.2)	173 (28.2)	0.676	0.023
Dyslipidemia, *N* (%)	3,208(44.9)	408 (66.4)	<0.001	0.444	828(67.4)	408(66.4)	0.713	0.021
HF, *N* (%)	549 (7.7)	64 (10.4)	0.020	0.095	131 (10.7)	64 (10.4)	0.936	0.008
CAD, *N* (%)	623 (8.7)	114 (18.6)	<0.001	0.290	216 (17.6)	114 (18.6)	0.652	0.025
PAOD, *N* (%)	100 (1.4)	11 (1.8)	0.545	0.031	24 (2.0)	11 (1.8)	0.952	0.012
Stroke, *N* (%)	452 (6.3)	46 (7.5)	0.298	0.046	111 (9.0)	46 (7.5)	0.302	0.056
CKD, *N* (%)	1,103 (15.5)	107 (17.4)	0.216	0.053	236 (19.2)	107 (17.4)	0.386	0.046
HCM, *N* (%)	97 (1.4)	9 (1.5)	0.970	0.009	18 (1.5)	9 (1.5)	>0.999	<0.001
Aspirin, *N* (%)	501 (7.0)	197 (32.1)	<0.001	0.666	374(30.5)	197 (32.1)	0.510	0.035
P_2_Y_12_ inhibitor, *N* (%)	191 (2.7)	74 (12.1)	<0.001	0.365	126 (10.3)	74 (12.1)	0.278	0.057
Warfarin, *N* (%)	29 (0.4)	5 (0.8)	0.250	0.052	11 (0.9)	5 (0.8)	>0.999	0.009
NOAC, *N* (%)	13 (0.2)	1 (0.2)	1.000	0.005	3 (0.2)	1 (0.2)	>0.999	0.018
ACEi/ARB, *N* (%)	442 (6.2)	151 (24.6)	<0.001	0.527	299 (24.3)	151 (24.6)	0.954	0.006

*AAD, antiarrhythmic drug; ACEi/ARB, angiotensin-converting enzyme inhibitor/angiotensin receptor blocker; AF, atrial fibrillation; CAD, coronary artery disease; CKD, chronic kidney disease; DM, diabetes mellitus; HCM, hypertrophic cardiomyopathy; HF, heart failure; HTN, hypertension; NOAC, novel oral anticoagulant; PAOD, peripheral arterial occlusive disease; PAC, premature atrial complex; SMD, standardized mean difference; Tx, treatment*.

### Long-Term Prognosis in Patients With High PAC Burden

**Figure 2:**
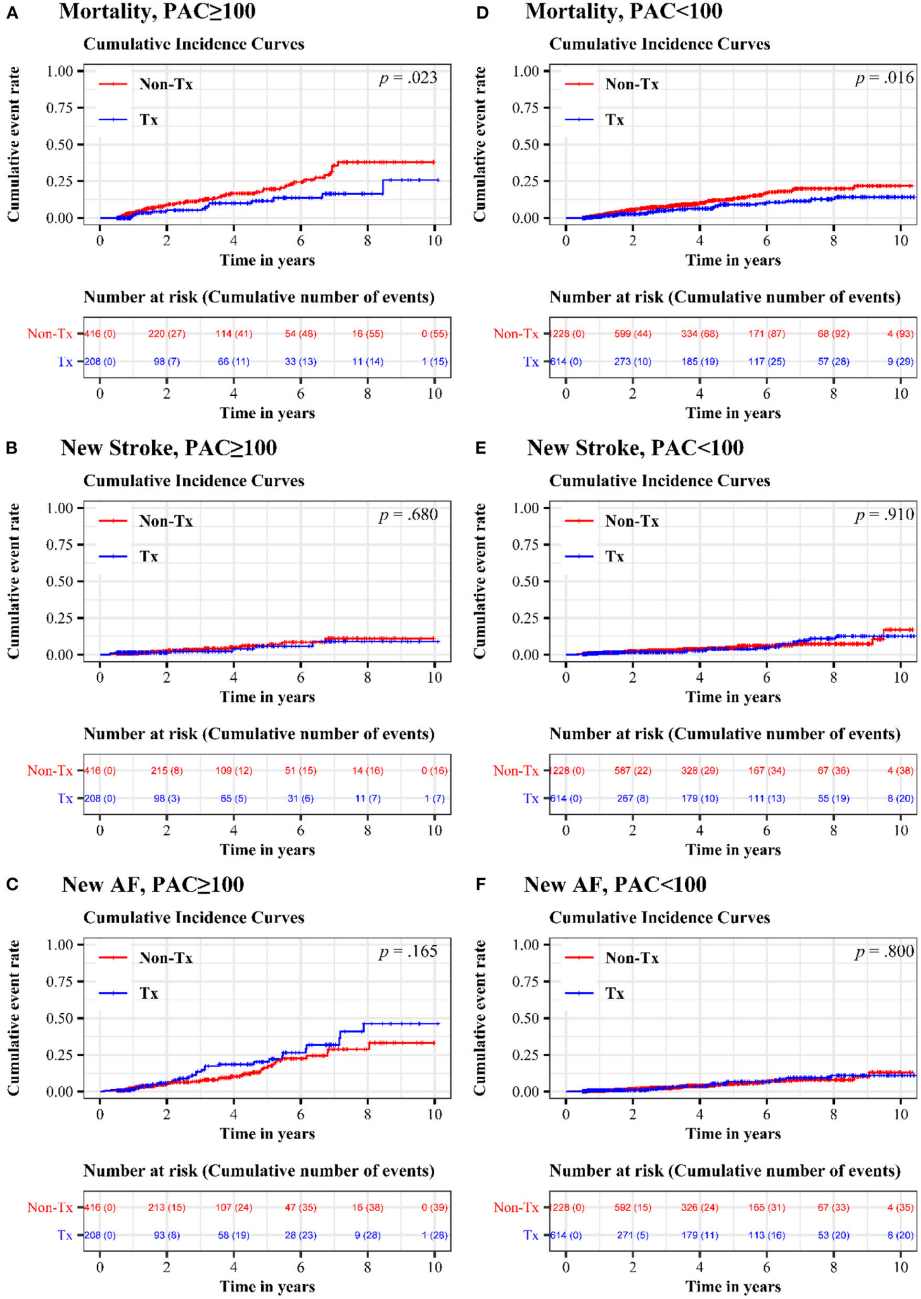
Cumulative incidence of mortality, new stroke, and new AF in treatment and non-treatment groups. In the high-burden sub-cohort, comparison of **(A)** long-term all-cause mortality **(B)** long-term cumulative new stroke rate and **(C)** long-term cumulative new onset rates of AF were exhibited. In the low-burden sub-cohort, **(D)** long-term all-cause mortality **(E)** long-term cumulative new stroke rate and **(F)** long-term cumulative new onset rates of AF were shown. AF, atrial fibrillation; PAC, premature atrial complex.

Figure 2 shows the 10-year cumulative incidence of each endpoint in patients with high and low PAC burdens after propensity score matching. Compared to patients who never or seldomly used beta blockers, patients in the treatment group had 48% risk reduction in long-term all-cause mortality (Figure 2A and Table 3, HR = 0.521, 95% CI = 0.294–0.923, *p* = 0.025). No significant difference in long-term cumulative new stroke rate was found between these two groups (Figure 2B and Table 3, HR = 0.830, 95% CI = 0.341–2.020, *p* = 0.681), as was also the case for long-term cumulative new onset rates of AF (Figure 2C and Table 3, HR = 1.410, 95% CI = 0.867–2.292, *p* = 0.167).

**Table 3:** Endpoint hazard ratios in high-burden and low-burden PAC sub-cohorts.

**Endpoint**	**HR (95% CI; *p*), high burden**	**HR (95% CI; *p*), low burden**
Mortality	0.521 (0.294, 0.923; *p* = 0.025)	0.601 (0.396, 0.913; *p* = 0.017)
New stroke	0.830 (0.341, 2.020; *p* = 0.681)	0.969 (0.562, 1.670; *p* = 0.910)
New AF	1.410 (0.867, 2.292; *p* = 0.167)	1.074 (0.619, 1.863; *p* = 0.800)

*AF, atrial fibrillation; CI, confidence interval; HR, hazard ratio*.

### Long-Term Prognosis in Patients With Low Burdens of PACs

Regular beta blocker use was associated with up to 40% risk reduction in long-term all-cause mortality (Figure 2D and Table 3, HR = 0.601, 95% CI = 0.396–0.913, *p* = 0.017), but no significant differences between the treatment and non-treatment groups in new onset of stroke (Figure 2E and Table 3, HR = 0.969, 95% CI = 0.562–1.670, *p* = 0.910) and new onset of AF (Figure 2F and Table 3, HR = 1.074, 95% CI = 0.619–1.863, *p* = 0.800) were noted.

### Subgroup Analysis

**Figure 3:**
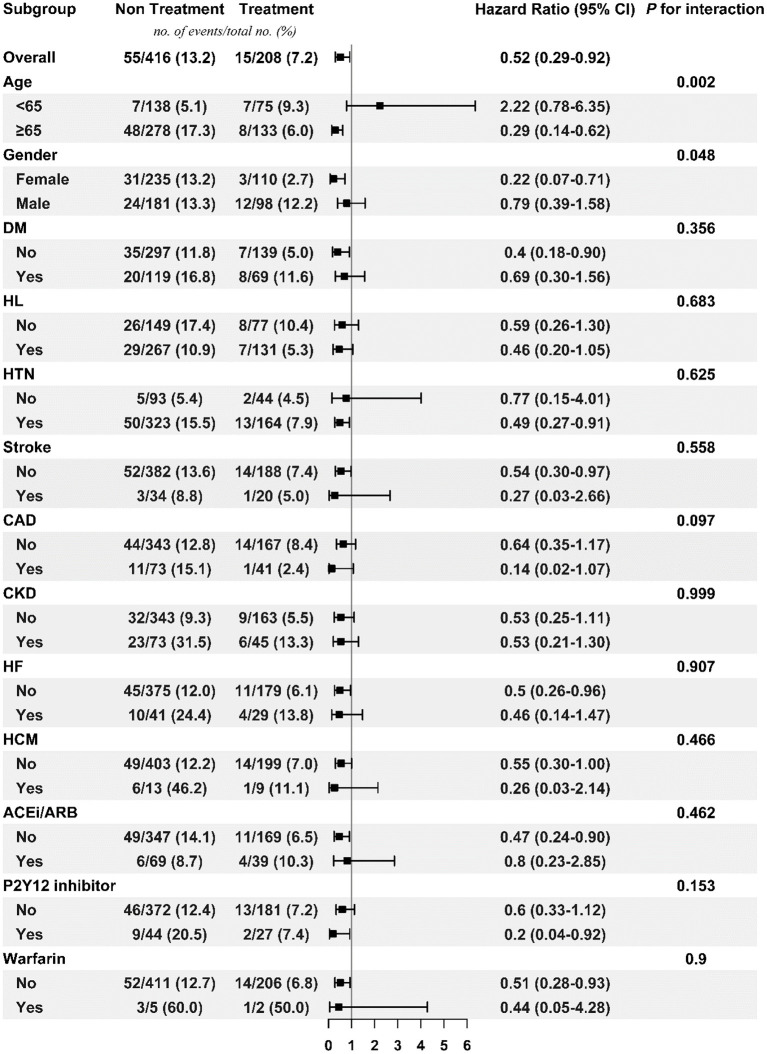
Subgroup analysis of treatment effect on mortality in the high PAC burden sub-cohort. ACEi/ARB, angiotensin-converting enzyme inhibitor/angiotensin receptor blocker; CAD, coronary artery disease; CKD, chronic kidney disease; DM, diabetes mellitus; HCM, hypertrophic cardiomyopathy; HF, heart failure; HL, hyperlipidemia; HTN, hypertension; PAC, premature atrial complex.

**Figure 4:**
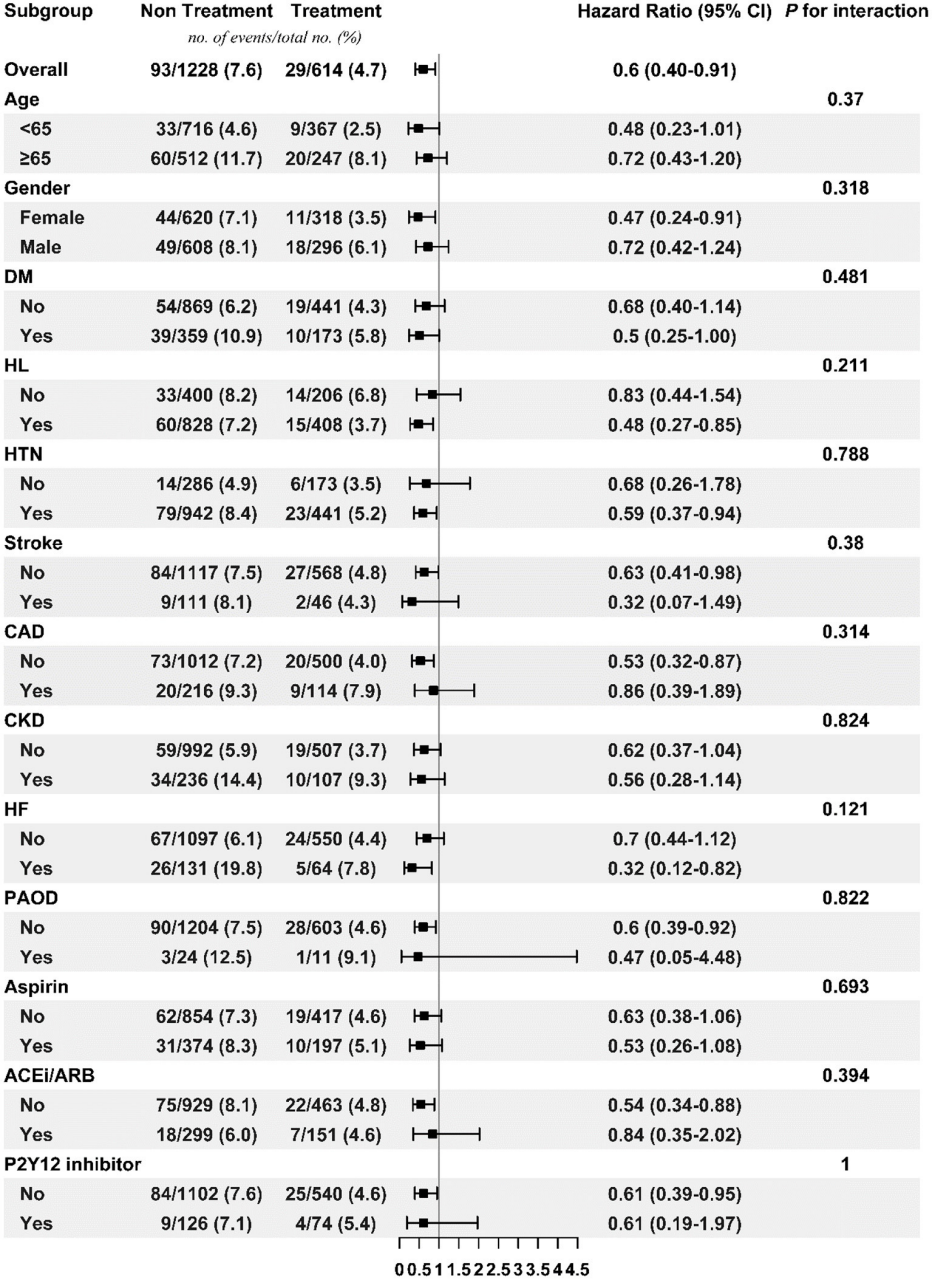
Subgroup analysis of treatment effect on mortality in the low PAC burden sub-cohort. ACEi/ARB, angiotensin-converting enzyme inhibitor/angiotensin receptor blocker; CAD, coronary artery disease; CKD, chronic kidney disease; DM, diabetes mellitus; HF, heart failure; HL, hyperlipidemia; HTN, hypertension; PAC, premature atrial complex; PAOD, peripheral arterial occlusive disease.

Figures 3, 4 respectively show the beneficial effects of beta blockers on all-cause mortality in patients with high or low burdens of PACs across the overall sub-cohort and pre-specified subgroups. Regardless of PAC burden in patients, treatment with beta blockers did not provide better outcomes in terms of new-onset stroke or AF over non-treated patients in each pre-specified subgroup (Supplementary Figures 1–4).

### Interval Change of PAC Burdens and Beta Blockers Regimen in Treatment Group

In our Supplementary Table 1, we presented the mean difference of PAC burdens among patients after PSM undergoing followed up Holter monitoring. Irrespective of treatment with beta blockers, the PAC burden among the high-burden sub-group was reduced, instead of lower-burden sub-group. Supplementary Figures 5, 6 exhibited respectively the proportion of different beta-blocker prescriptions in treatment group, and the mean daily dosage of beta blockers. The most common prescription was bisoprolol (mean dosage: 3.8 ± 1.6 mg) followed by propranolol (mean dosage: 22.1 ± 13.5 mg).

## Discussion

In this long-term follow-up study, we used propensity score matching to show that beta blocker treatment can lower all-cause mortality in patients with high or low PAC burdens. However, no difference in new onset of stroke or AF between treatment and non-treatment groups was found. To the best of our knowledge, this study is the first study to elucidate the beneficial effects of beta blockers on the long-term prognosis of patients with PACs, which are considered to be key risk factors for all-cause mortality and major cardiovascular adverse events (2–6, 8). Although strong evidence is still lacking, the latest consensus document by the European Heart Rhythm Association (9) suggests discussing the initiation of oral anticoagulants for the prevention of stroke with patients that have a high PAC burden (>500 PACs per 24 h or any episode of runs >20 PACs). This suggestion is based on the dose-response effect of PACs on the risk of AF (10). However, besides oral anticoagulation, the treatment of patients with high PAC burdens remains an important unmet clinical need.

Beta blockers targeting the autonomic nervous system are well-known for their efficacy in improving symptoms, reducing hospitalizations, and/or prolonging survival for heart failure patients in randomized controlled studies (11–14). PACs share the same main feature as heart failure (e.g. sympathetic overactivation) (7), and thus in our daily practice, beta blockers are commonly prescribed to treat symptomatic patients with PACs. It is reasonable to explore their effects beyond symptom control. In this study, treatment with beta blockers significantly decreased the risk of all-cause mortality across patients with high or low PAC burdens. Although decreased mortality in the treatment group may be partially due to the well-known benefits of beta blockers in heart failure and sudden cardiac death, our study also demonstrated the consistent benefits in lowering mortality rate of patients without heart failure in the treatment group, as shown in Figures 3, 4. While concerning similar but statistically significant difference in PVC burdens between treatment and non-treatment groups in low-burden PAC sub-cohort, in our recent data (15), moderate (1,000–10,000 beats per 24 h) and high burdens (>10,000 beats per 24 h) of PVCs had higher risk of cardiovascular death than low-burden PVC group (Find and Gray's competing risk model adjusted HR = 1.48, 95% CI = 1.09–2.01, *p* < 0.05; HR = 1.70, 95% CI = 1.06–2.71, *p* < 0.05). However, our data showed no difference in all-cause mortality. Our current study focused on the all-cause mortality, but not cardiovascular death in PAC patients taking beta blockers. We believed that this difference would not affect our main results.

The benefits of beta blockers were not apparent in the prevention of new-onset stroke for both the high-burden and low-burden sub-cohorts, and the existing data regarding this is somewhat mixed; Ziff et al. previously reported that compared with a placebo group, the risk of stroke decreased in hypertensive patients treated with beta blockers, but beta blockers were less effective than angiotensin converting enzyme inhibitors/angiotensin receptor blockers and calcium channel blockers (16). No benefits of stroke secondary prevention for patients in the acute phase after stroke (17), with coronary artery disease, or who required cardiac or non-cardiac surgery were noted (16).

The main pathophysiology of PACs as precursors of AF has been attributed to enhanced automaticity or trigger activity related to adrenergic overactivation (7). Regional autonomic modulation to decrease sympathetic outflow may be effective in preventing atrial arrhythmia. Systemic beta blockers have been proven to prevent AF occurrence or recurrence, and continuous treatment with metoprolol CR starting at least one week before direct current cardioversion has been shown to be effective in maintaining sinus rhythm at 6 months after cardioversion (18). Prophylactic beta blockers to prevent postoperative AF is a well-established practice that should be started or continued before cardiac surgery (19). As mentioned in the latest ESC AF guideline (20), some small studies showed the benefits in preventing AF occurrence or recurrence (18, 21); however, most evidence were against a significant role of beta-blockers in preventing AF (22). The observational beneficial effects were derived from clinically significant and symptomatic AF to silent AF, by the effect of rate control from beta-blockers. Similarly, this study showed that regardless of high or low PAC burden, there was no difference in the incidence of new onset AF between treatment and non-treatment groups. Another possibility is that this may be because patients are mostly asymptomatic or already familiar with PAC-related symptoms, and therefore fewer electrocardiography or 24-h Holter monitor tests would be requested, thereby lessening the chance of detecting AF in such patients during follow-up.

In the high burden population younger than 65 years old, beta blockers significantly increased the risk of new-onset AF and were less effective on mortality prevention. In fact, several possibilities were hypothesized: first, these patients were very symptomatic and thus took regular beta blockers and presumed followed up Holter or ECG studies were arranged more frequently, and thus more AF episodes were detected. Another possible reason was that, as mentioned above, beta blockers could not significantly prevent AF occurrence. Finally, although the incidence rates of new-onset AF between high burden and low burden sub-cohort were not directly compared, it was interesting to note that higher event rate in high burden than low burden sub-cohort irrespective of treatment. Adrenergic overactivation was that overwhelmed in this young subgroup, and more AF indeed occurred and worsened the long-term prognosis.

### Limitations

Because of the retrospective nature of this study, many patients did not do follow-up Holter monitoring after receiving medication, and thus it is uncertain whether greater reduction in PAC burden or improvement in heart rate variability would be associated with better outcomes. In addition, as mentioned above, new-onset AF may be underestimated in this cohort, perhaps due to a high prevalence of asymptomatic patients or increased familiarity with arrhythmia-related symptoms, resulting in fewer test requests and under-detection of AF. Finally, it was not possible to determine if patients were symptomatic or not in this study, and therefore it is unknown whether the effects of beta blockers are consistent across symptomatic and asymptomatic patients.

## Conclusions

In this retrospective long-term propensity-matched cohort study, beta blocker usage was associated with lower all-cause mortality in patients with baseline high or low PAC burdens. Interestingly, this effect was not directly associated with reduction of new-onset stroke or AF, and further research to identify the underlying mechanism(s) is warranted.

## Data Availability Statement

The raw data supporting the conclusions of this article will be made available by the authors, without undue reservation.

## Ethics Statement

The studies involving human participants were reviewed and approved by Institutional Review Board, National Cheng Kung University Hospital. Written informed consent for participation was not required for this study in accordance with the national legislation and the institutional requirements.

## Author Contributions

T-CH contributed to conception and design of the study and wrote the first draft of the manuscript. T-CH, P-TL, and M-SH conducted data collection and cleaning from the Databank. P-HC performed the statistical analysis. P-FS conducted statistical validation. T-CH and P-HC prepared the figures and tables. P-FS and P-YL edited and reviewed the manuscript. All authors reviewed, edited, and approved of the final submitted manuscript.

## Funding

This study was supported by grants 109-2634-F-006-020 and 110-2634-F-006-020 from the Ministry of Science and Technology of Taiwan, and grants D108-G2512, D109-G4803, D109-G4804, D109-G2512, and D110-G2512 from the Higher Education Sprout Project of the Ministry of Education of Taiwan, to the Headquarters of University Advancement at National Cheng Kung University.

## Conflict of Interest

The authors declare that the research was conducted in the absence of any commercial or financial relationships that could be construed as a potential conflict of interest.

## Publisher's Note

All claims expressed in this article are solely those of the authors and do not necessarily represent those of their affiliated organizations, or those of the publisher, the editors and the reviewers. Any product that may be evaluated in this article, or claim that may be made by its manufacturer, is not guaranteed or endorsed by the publisher.

## References

[1] Conen D Adam M Roche F Barthelemy JC Felber Dietrich D Imboden M. Premature atrial contractions in the general population: frequency and risk factors. Circulation. (2012) 126:2302–8. 10.1161/CIRCULATIONAHA.112.112300 23048073

[2] Huang TC Lee PT Huang MS Su PF Liu PY. Higher premature atrial complex burden from the Holter examination predicts poor cardiovascular outcome. Sci Rep. (2021) 11:12198. 10.1038/s41598-021-91800-4 34108588 PMC8190115

[3] Larsen BS Kumarathurai P Falkenberg J Nielsen OW Sajadieh A. Excessive atrial ectopy and short atrial runs increase the risk of stroke beyond incident atrial fibrillation. J Am Coll Cardiol. (2015) 66:232–41. 10.1016/j.jacc.2015.05.018 26184616

[4] Binici Z Intzilakis T Nielsen OW Køber L Sajadieh A. Excessive supraventricular ectopic activity and increased risk of atrial fibrillation and stroke. Circulation. (2010) 121:1904–11. 10.1161/CIRCULATIONAHA.109.874982 20404258

[5] Lin CY Lin YJ Chen YY Chang SL Lo LW Chao TF. Prognostic significance of premature atrial complexes burden in prediction of long-term outcome. J Am Heart Assoc. (2015) 4:e002192. 10.1161/JAHA.115.002192 26316525 PMC4599506

[6] Murakoshi N Xu D Sairenchi T Igarashi M Irie F Tomizawa T. Prognostic impact of supraventricular premature complexes in community-based health checkups: the Ibaraki Prefectural Health Study. Eur Heart J. (2015) 36:170–8. 10.1093/eurheartj/ehu407 25358506

[7] Chen PS Chen LS Fishbein MC Lin SF Nattel S. Role of the autonomic nervous system in atrial fibrillation: pathophysiology and therapy. Circ Res. (2014) 114:1500–15. 10.1161/CIRCRESAHA.114.303772 24763467 PMC4043633

[8] Himmelreich JCL Lucassen WAM Heugen M Bossuyt PMM Tan HL Harskamp RE. Frequent premature atrial contractions are associated with atrial fibrillation, brain ischaemia, and mortality: a systematic review and meta-analysis. Europace. (2019) 21:698–707. 10.1093/europace/euy276 30508087

[9] Arnar DO Mairesse GH Boriani G Calkins H Chin A Coats A. Management of asymptomatic arrhythmias: a European Heart Rhythm Association (EHRA) consensus document, endorsed by the Heart Failure Association (HFA), Heart Rhythm Society (HRS), Asia Pacific Heart Rhythm Society (APHRS), Cardiac Arrhythmia Society of Southern Africa (CASSA), and Latin America Heart Rhythm Society (LAHRS). Europace. (2019) euz046. 10.1093/europace/euz046 30882141

[10] Gladstone DJ Dorian P Spring M Panzov V Mamdani M Healey JS. Atrial premature beats predict atrial fibrillation in cryptogenic stroke: results from the EMBRACE trial. Stroke. (2015) 46:936–41. 10.1161/STROKEAHA.115.008714 25700289

[11] CIBIS Investigators and Committees. A randomized trial of beta-blockade in heart failure. The Cardiac Insufficiency Bisoprolol Study (CIBIS). CIBIS Investigators and Committees. Circulation. (1994) 90:1765–73. 10.1161/01.CIR.90.4.1765 7923660

[12] CIBIS-II Investigators and Committees. The cardiac insufficiency bisoprolol study II (CIBIS-II): a randomised trial. Lancet. (1999) 353:9–13. 10.1016/S0140-6736(98)11181-9 10023943

[13] MERIT-HF Study Group. Effect of metoprolol CR/XL in chronic heart failure: Metoprolol CR/XL Randomised Intervention Trial in Congestive Heart Failure (MERIT-HF). Lancet. (1999) 353:2001–7. 10.1016/S0140-6736(99)04440-2 10376614

[14] Packer M Coats AJ Fowler MB Katus HA Krum H Mohacsi P. Effect of carvedilol on survival in severe chronic heart failure. N Engl J Med. (2001) 344:1651–8. 10.1056/NEJM200105313442201 11386263

[15] Lee PT Huang TC Huang MC Hsu LW Su PF Liu YW. Burden of ventricular premature complex is associated with cardiovascular mortality. Front Cardiovasc Med. (2022) 8:797976. 10.3389/fcvm.2021.797976 35187109 PMC8850345

[16] Ziff OJ Samra M Howard JP Bromage DI Ruschitzka F Francis DP. Beta-blocker efficacy across different cardiovascular indications: an umbrella review and meta-analytic assessment. BMC Med. (2020) 18:103. 10.1186/s12916-020-01564-3 32366251 PMC7199339

[17] Balla HZ Cao Y Ström JO. Effect of beta-blockers on stroke outcome: a meta-analysis. Clin Epidemiol. (2021) 13:225–36. 10.2147/CLEP.S268105 33762851 PMC7982440

[18] Nergårdh AK Rosenqvist M Nordlander R Frick M. Maintenance of sinus rhythm with metoprolol CR initiated before cardioversion and repeated cardioversion of atrial fibrillation: a randomized double-blind placebo-controlled study. Eur Heart J. (2007) 28:1351–7. 10.1093/eurheartj/ehl544 17329409

[19] Dobrev D Aguilar M Heijman J Guichard JB Nattel S. Postoperative atrial fibrillation: mechanisms, manifestations and management. Nat Rev Cardiol. (2019) 16:417–36. 10.1038/s41569-019-0166-5 30792496

[20] Hindricks G Potpara T Dagres N Arbelo E Bax JJ Blomström-Lundqvist C. 2020 ESC Guidelines for the diagnosis and management of atrial fibrillation developed in collaboration with the European Association for Cardio-Thoracic Surgery (EACTS): The Task Force for the diagnosis and management of atrial fibrillation of the European Society of Cardiology (ESC) Developed with the special contribution of the European Heart Rhythm Association (EHRA) of the ESC. Eur Heart J. (2020) 42:373–498. 10.1093/eurheartj/ehab648 32860505

[21] Capucci A Botto G Molon G Spampinato A Favale S Proclemer A. The drug and pace health clinical evaluation (DAPHNE) study: a randomized trial comparing sotalol versus beta-blockers to treat symptomatic atrial fibrillation in patients with brady-tachycardia syndrome implanted with an antitachycardia pacemaker. Am Heart J. (2008) 156:373.e1–8. 10.1016/j.ahj.2008.01.032 18657671

[22] Lafuente-Lafuente C Valembois L Bergmann JF Belmin J. Antiarrhythmics for maintaining sinus rhythm after cardioversion of atrial fibrillation. Cochrane Database Syst Rev. (2015) 3:CD005049. 10.1002/14651858.CD005049.pub4 25820938

