# Supplementary material for: The Beneficial Effects of Beta Blockers on the Long-Term Prognosis of Patients With Premature Atrial Complexes
Source: Front Cardiovasc Med. 2022 Feb 16;9:806743. doi: 10.3389/fcvm.2022.806743 (PMC8890474; doi:10.3389/fcvm.2022.806743)
Supplement: Supplementary file 1 [file Data_Sheet_1.docx]

Supplementary Material





**Supplementary Figure 1.** Subgroup analysis of treatment effect on new stroke in the high PAC burden sub-cohort. ACEi/ARB, angiotensin-converting enzyme inhibitor/angiotensin receptor blocker; CAD, coronary artery disease; CKD, chronic kidney disease; DM, diabetes mellitus; HF, heart failure.





**Supplementary Figure 2.** Subgroup analysis of treatment effect on new AF in the high PAC burden sub-cohort. ACEi/ARB, angiotensin-converting enzyme inhibitor/angiotensin receptor blocker; AF, atrial fibrillation; CAD, coronary artery disease; CKD, chronic kidney disease; DM, diabetes mellitus; HCM, hypertrophic cardiomyopathy; HF, heart failure; HL, hyperlipidemia; HTN, hypertension.





**Supplementary Figure 3.** Subgroup analysis of treatment effect on new stroke in the low PAC burden sub-cohort. ACEi/ARB, angiotensin-converting enzyme inhibitor/angiotensin receptor blocker; CAD, coronary artery disease; CKD, chronic kidney disease; DM, diabetes mellitus; HCM, hypertrophic cardiomyopathy; HF, heart failure; HL, hyperlipidemia; HTN, hypertension.





**Supplementary Figure 4.** Subgroup analysis of treatment effect on new AF in the low PAC burden sub-cohort. ACEi/ARB, angiotensin-converting enzyme inhibitor/angiotensin receptor blocker; AF, atrial fibrillation; CAD, coronary artery disease; CKD, chronic kidney disease; DM, diabetes mellitus; HCM, hypertrophic cardiomyopathy; HF, heart failure; HL, hyperlipidemia.


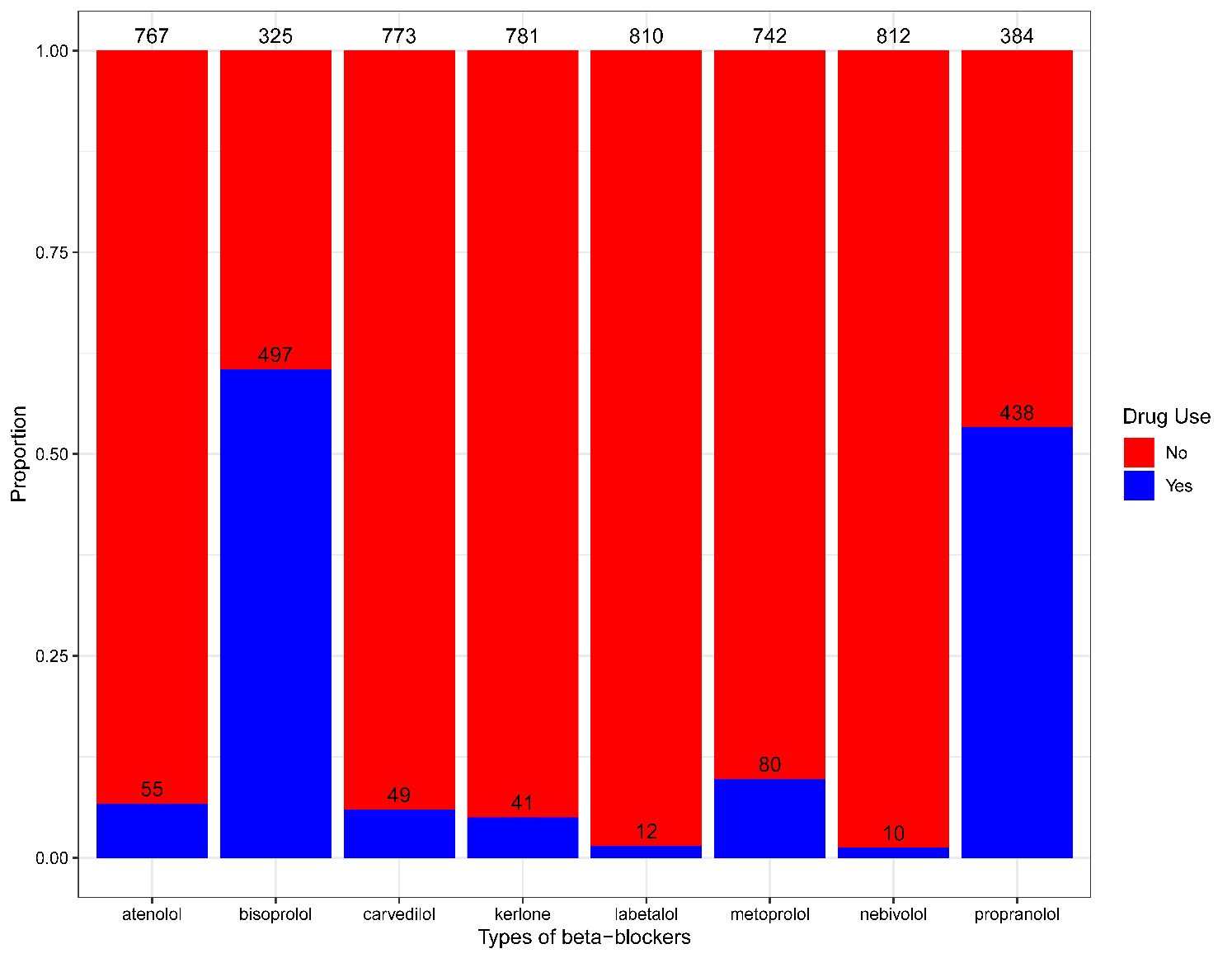


**Supplementary Figure 5.** The proportion of different beta-blocker prescriptions in treatment group.


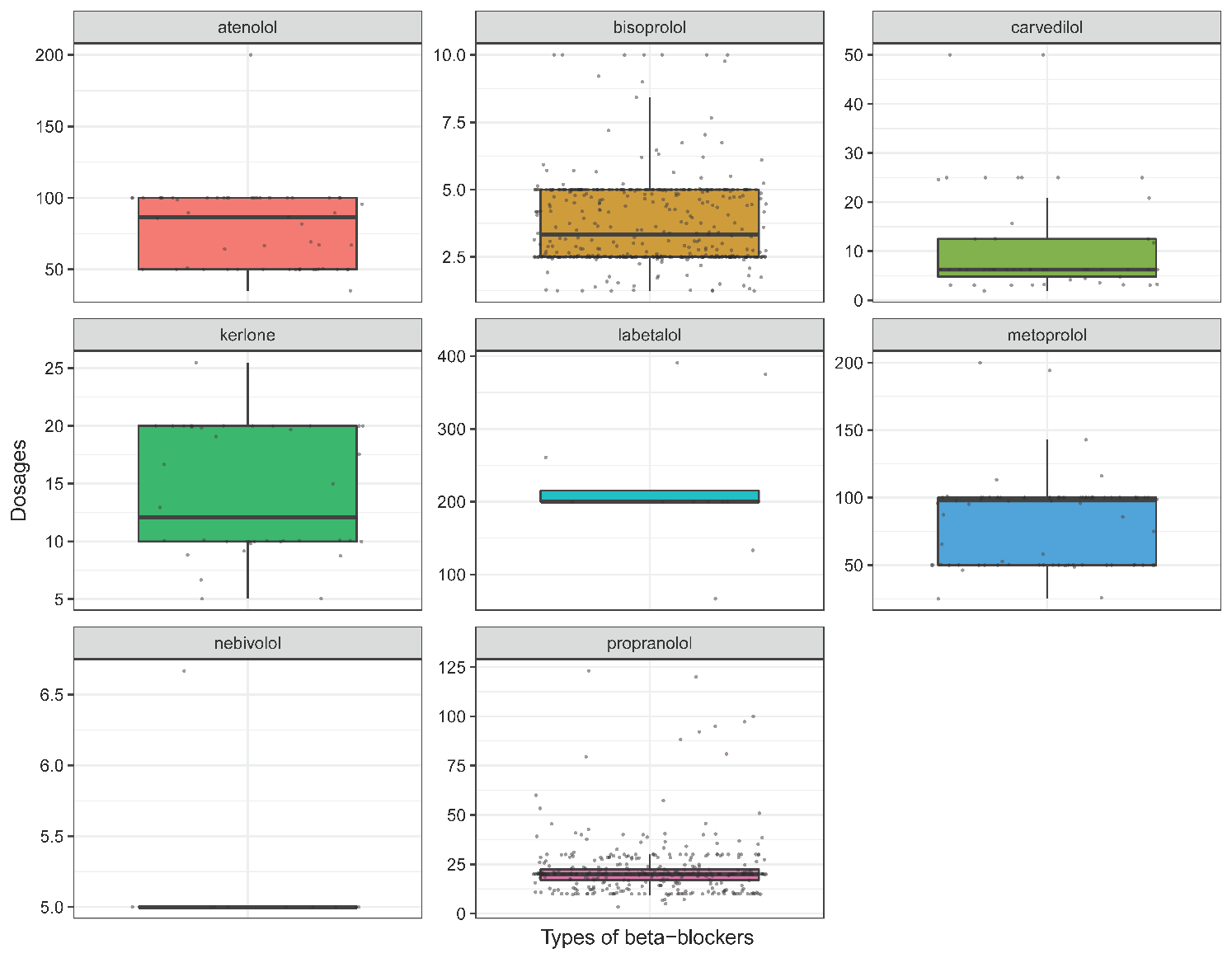


**Supplementary Figure 6.** Mean daily dosage of beta-blocker prescriptions

**Supplementary Table 1.** PAC burdens in the first and latest Holter monitoring of patients after propensity score matching

|  | High-burden non-Tx  (N = 68) | | | High-burden Tx  (N = 29) | | | Low-burden non-Tx  (N = 165) | | | Low-burden Tx  (N = 84) | | |
| --- | --- | --- | --- | --- | --- | --- | --- | --- | --- | --- | --- | --- |
|  | First | Latest | *p* | First | Latest | *p* | First | Latest | *p* | First | Latest | *p* |
| PAC, mean (SD) | 3076.5  (6653.3) | 2230.2  (6937.3) | 0.009 | 7256.6  (12422.2) | 1959.8  (5814.5) | 0.031 | 23.0  (22.4) | 367.3  (1904.9) | < 0.001 | 19.2  (21.7) | 840.1  (4178.3) | < 0.001 |
